# Considerações sobre o Diagnóstico e Prognóstico do Linfoma Cardíaco Primário

**DOI:** 10.36660/abc.20250521

**Published:** 2025-12-11

**Authors:** Thalisson Gonçalves Almeida, Tales Gabriel Ferro de Oliveira, Gustavo da Silva Soares, Pedro Pereira Tenório

**Affiliations:** 1 Universidade Federal do Vale do São Francisco Paulo Afonso BA Brasil Universidade Federal do Vale do São Francisco (UNIVASF), Paulo Afonso, BA – Brasil

**Keywords:** Linfoma Cardíaco, Diagnóstico por Imagem, Relato de Caso, Cardio-Oncologia

**Prezado Editor,**

Gostaríamos de parabenizar os autores pelo relevante relato de caso "Linfoma Cardíaco: Um Relato de Caso", publicado nos Arquivos Brasileiros de Cardiologia (ABC Cardiol).^1^ O artigo aborda uma condição clínica rara e desafiadora, o linfoma cardíaco primário (LCP), que, como bem destacado no artigo, é um tumor agressivo com prognóstico ruim e sintomas inespecíficos, o que dificulta o diagnóstico precoce.^1^ Entre os pontos fortes do artigo, destacamos a raridade do caso, por se tratar de linfoma difuso de grandes células B (LDGC) com envolvimento cardíaco primário, dada a baixa incidência desses tumores (aproximadamente 0,04% dos tumores cardíacos primários malignos, com linfomas representando cerca de 1,3% destes) sendo o LDGC o subtipo mais comum, frequentemente localizado nas câmaras direitas do coração.^2 , 3^

A documentação detalhada de um caso tão incomum contribui significativamente para a literatura médica, sobretudo devido à sua raridade e aos sintomas inespecíficos que podem mimetizar outras condições como insuficiência cardíaca (IC), o diagnóstico do LCP é complexo. Um relato de caso com descrição minuciosa dos achados de imagem — ecocardiograma, angiotomografia, ressonância magnética cardíaca — e confirmação histopatológica, serve como um guia valioso para médicos que se deparam com massas cardíacas atípicas.

Casos clínicos detalhados são ferramentas pedagógicas essenciais para estudantes e residentes, ajudando-os a reconhecer, investigar e gerenciar doenças raras que não são abordadas extensivamente em livros-texto ou aulas. Além disso, incentivam o desenvolvimento acadêmico e o pensamento científico, como destacam Florek e Dellavalle,^4^ sendo frequentemente a primeira experiência de escrita científica para estudantes. Além disso, Danish et al.^5^ enfatizam que os relatos de caso podem apresentar casos novos e raros de maneira que levem à expansão da pesquisa e à geração de novas hipóteses inovadoras.

O artigo descreve a evolução clínica do paciente, as complicações pós-operatórias (como edema agudo de pulmão e necessidade de marca-passo) e a resposta ao tratamento quimioterápico, incluindo a intercorrência de hemorragia digestiva e a recaída da doença, além de explorar a importância de exames de imagem não invasivos, como o ecocardiograma transtorácico e, principalmente, a ressonância magnética cardiovascular (RMC) para a identificação e caracterização das massas cardíacas.^1^

O tratamento com R-CHOP, esquema padrão para LDGC, demonstrou benefício terapêutico com taxa de remissão de até 70%, embora a presença de antraciclinas represente risco significativo de cardiotoxicidade, exigindo acompanhamento rigoroso.^6^ Ao apresentar a descrição do tratamento, incluindo a ressecção cirúrgica do tumor com reconstrução de veia cava superior e rafia do átrio direito, e a subsequente quimioterapia com o esquema R-CHOP, o artigo de Pamplona et al.,^1^ 2025 demonstra a complexidade do manejo desses pacientes. A discussão das complicações pós-operatórias, como bradicardia juncional, fibrilação atrial e edema agudo de pulmão, além da intercorrência de hemorragia digestiva durante o tratamento, ressalta os desafios clínicos enfrentados. A conclusão do artigo, que reforça a necessidade de incluir o LCP como diagnóstico diferencial de massas cardíacas, é crucial. O diagnóstico precoce pode melhorar significativamente o prognóstico e aumentar a sobrevida dos pacientes, permitindo um encaminhamento rápido para tratamento específico.

No entanto, alguns pontos poderiam ser considerados para aprimoramento. O artigo menciona que o paciente optou por participar de um ensaio clínico para testar um novo quimioterápico e que a evolução do paciente não pode ser divulgada. Embora compreensível por questões éticas e de confidencialidade de ensaios clínicos, a falta de informações sobre o desfecho final do paciente limita a compreensão completa da eficácia do tratamento a longo prazo e do prognóstico do caso específico. Diretrizes como a CARE (CAse REport) recomendam a apresentação completa do seguimento, incluindo resposta ao tratamento, efeitos adversos e prognóstico, elementos que enriquecem o relato e oferecem subsídios importantes à prática médica.^7 - 9^

Para um relato de caso, a evolução completa é um elemento importante para extrair futuras lições. As complicações pós-operatórias e durante o tratamento quimioterápico (edema agudo de pulmão, necessidade de marcapasso, hemorragia digestiva) são mencionadas. Uma discussão um pouco mais detalhada sobre a possível relação dessas intercorrências com o tipo de tumor, o tratamento, ou fatores específicos do paciente, poderia enriquecer o caso. Por exemplo, a colite infecciosa que levou à hemorragia digestiva, um evento grave, poderia ter uma breve reflexão sobre manejo ou prevenção em pacientes oncológicos sob tratamento quimioterápico, o que tornaria o caso ainda mais valioso para os leitores.

Em suma, o presente relato de caso é uma valiosa adição ao conhecimento sobre o LCP, destacando os desafios diagnósticos e terapêuticos. As observações acima são sugestões para potencializar ainda mais o impacto e a completude de futuros relatos de caso sobre condições tão complexas.
