# Supplementary material for: Considerações sobre o Diagnóstico e Prognóstico do Linfoma Cardíaco Primário
Source: Arq Bras Cardiol. 2025 Dec 11;122(10):e20250521. [Article in Portuguese] doi: 10.36660/abc.20250521 (PMC12711226; doi:10.36660/abc.20250521)
Supplement: Translation

# Translation: Considerations on the Diagnosis and Prognosis of Primary Cardiac Lymphoma

**DOI:** 10.36660/abc.20250521i

**Authors:** Thalisson Gonçalves Almeida, Tales Gabriel Ferro de Oliveira, Gustavo da Silva Soares, Pedro Pereira Tenório

**Affiliations:** 1 Paulo Afonso BA Brazil Universidade Federal do Vale do São Francisco (UNIVASF), Paulo Afonso, BA – Brazil

**Keywords:** Cardiac Lymphoma, Imaging Diagnosis, Case Report, Cardio-Oncology

**Dear Editor,**

We would like to congratulate the authors on their relevant case report, "Cardiac Lymphoma: A Case Report," published in the *Arquivos Brasileiros de Cardiologia* (ABC Cardiol).^1^The article addresses a rare and challenging clinical condition, primary cardiac lymphoma (PCL), which, as highlighted in the article, is an aggressive tumor with a poor prognosis and nonspecific symptoms, making early diagnosis difficult.^1^Among the article's strengths, we highlight the rarity of the case, as it is a diffuse large B-cell lymphoma (DLBCL) with primary cardiac involvement, given the low incidence of these tumors (approximately 0.04% of primary malignant cardiac tumors, with lymphomas representing about 1.3% of these), with DLBCL being the most common subtype, frequently located in the right chambers of the heart.^2 , 3^

The detailed documentation of such an unusual case contributes significantly to the medical literature, especially due to its rarity and the nonspecific symptoms that can mimic other conditions, such as heart failure (HF), making the diagnosis of PCL complex. A case report with a meticulous description of the imaging findings—echocardiogram, computed tomography angiography, cardiac magnetic resonance imaging—and histopathological confirmation serves as a valuable guide for physicians who encounter atypical cardiac masses.

Detailed clinical cases are essential pedagogical tools for students and residents, helping them to recognize, investigate, and manage rare diseases that are not extensively covered in textbooks or lectures. Furthermore, they encourage academic development and scientific thinking, as highlighted by Florek & Dellavalle,^4^often being the first scientific writing experience for students. In addition, Danish et al.^5^emphasize that case reports can present new and rare cases in a way that leads to the expansion of research and the generation of innovative new hypotheses.

The article describes the patient's clinical evolution, postoperative complications (such as acute pulmonary edema and the need for a pacemaker), and response to chemotherapy treatment, including the occurrence of gastrointestinal bleeding and disease relapse, as well as exploring the importance of non-invasive imaging exams, such as transthoracic echocardiography and, especially, cardiovascular magnetic resonance imaging (CMR) for the identification and characterization of cardiac masses.^1^

Treatment with R-CHOP, the standard regimen for DLBCL, has demonstrated therapeutic benefit with remission rates as high as 70%, although the presence of anthracyclines poses a significant risk of cardiotoxicity, requiring close monitoring.^6^In presenting the treatment description, including surgical resection of the tumor with reconstruction of the superior vena cava and repair of the right atrium, and subsequent chemotherapy with the R-CHOP regimen, the article by Pamplona et al.^1^demonstrates the complexity of managing these patients. The discussion of postoperative complications, such as junctional bradycardia, atrial fibrillation, and acute pulmonary edema, in addition to the occurrence of gastrointestinal bleeding during treatment, highlights the clinical challenges faced. The article's conclusion, which reinforces the need to include PCL as a differential diagnosis for cardiac masses, is crucial. Early diagnosis can significantly improve prognosis and increase patient survival, allowing for rapid referral for specific treatment.

However, some points could be considered for improvement. The article mentions that the patient chose to participate in a clinical trial to test a new chemotherapy drug and that the patient's progress cannot be disclosed While understandable due to ethical and confidentiality considerations in clinical trials, the lack of information on the patient's final outcome limits a complete understanding of the long-term effectiveness of treatment and the prognosis of the specific case. Guidelines such as CARE (CAse REport) recommend the complete presentation of follow-up, including treatment response, adverse effects, and prognosis, elements that enrich the report and offer important support to medical practice.^7 - 9^

For a case report, the complete evolution is an important element for drawing future lessons. Postoperative complications and those occurring during chemotherapy (acute pulmonary edema, need for a pacemaker, gastrointestinal bleeding) are mentioned. A slightly more detailed discussion of the possible relationship of these complications with the type of tumor, the treatment, or specific patient factors could enrich the case. For example, the infectious colitis that led to gastrointestinal bleeding, a serious event, could be briefly reflected upon in terms of management or prevention in cancer patients undergoing chemotherapy, which would make the case even more valuable for readers.

In summary, this case report is a valuable addition to the knowledge about PCL, highlighting the diagnostic and therapeutic challenges. The observations above are suggestions to further enhance the impact and completeness of future case reports on such complex conditions.
